# Arginine-mediated inhibition of macrophage apoptosis by *Escherichia coli* nissle 1917 in *Salmonella* typhimurium-induced intestinal inflammation

**DOI:** 10.3389/fimmu.2025.1684234

**Published:** 2025-10-16

**Authors:** Lu Zhang, Jiahui Yang, Qixue Shi, Yanyan Chu, Jiarui Cao, Chenglong Shang, Changlin Zhou, Lingman Ma

**Affiliations:** School of Life- Science and Technology, China-Pharmaceutical. University, Nanjing, Jiangsu, China

**Keywords:** intestinal inflammation, inhibition of macrophage apoptosis, Escherichia coli nissle 1917, arginine, Salmonella typhimurium infection

## Abstract

**Introduction:**

Intestinal inflammation is a chronic, relapsing disorder of the gastrointestinal tract characterized by dysregulated immune responses, microbial dysbiosis, and environmental influence. Pathogen clearance is related to the severity of intestinal diseases and macrophage apoptosis. Escherichia coli Nissle 1917 (EcN) alleviates the intestinal inflammation caused by Salmonella enterica serotype Salmonella Typhimurium (Salmonella Typhimurium, S. Tm) infection.

**Methods:**

Through gene editing, we found that the regulatory gene arcA largely affects arginine production in EcN via the arginine deiminase pathway. In vitro studies demonstrated that EcN alleviates S. Tm-induced apoptosis in RAW264.7 cells by enhancing intracellular arginine levels. Specifically, arginine generated by EcN can reduce S. Tm infection-induced generation of reactive oxygen species (ROS), chromatin condensation, DNA fragmentation, disruption of plasma membrane integrity, and decrease in mitochondrial membrane potential. Additionally, arginine administration in S. Tm-challenged mice decreased bacterial burden in the gut, suppressed Caspase-3 (CASPASE3) activation, mitigated both inflammation and apoptosis, and maintained epithelial barrier.

**Result and discussion:**

Mechanistically, arginine suppresses S. Tm-driven B-cell lymphoma-2 (BCL2) downregulation, inhibiting apoptosis. Further analysis revealed that arginine may disrupt the interaction between ribosomal protein S3 (RPS3) and serine/serine and arginine rich splicing factor 3 (SRSF3), thereby further suppressing the expression of apoptosis-related proteins induced by S. Tm. Our research offers new targets and approaches for treating bacterial infection-induced intestinal inflammation.

## Introduction

Intestinal inflammation is a chronic and recurrent inflammatory condition of the gastrointestinal tract (1, 2). The pathogenesis of intestinal inflammation involves intricate interactions between the gut immune system, the gut microbiome, and environmental factors (3).

The gut, recognized as the largest immune organ in the human body, harbors a diverse microbial community composed of bacteria, fungi, archaea, protozoa, and viruses, with bacteria constituting the predominant component (4, 5). Emerging evidence underscores the role of gut bacteria in modulating the host’s immune response. Notably, intestinal inflammation has been linked to gut dysbiosis, a condition characterizfed by reduced microbial diversity and an imbalance between symbiotic and pathogenic microorganisms (6, 7). Several bacterial pathogens have been implicated in the etiology of intestinal inflammation, including *Salmonella* (8), *Clostridium difficile* (9), *Campylobacter jejuni*, *Helicobacter pylori*, *Vibrio cholerae*, *Listeria monocytogenes*, and *Clostridium perfringens* (10). *Salmonella enterica* serovar Typhimurium (*Salmonella* Typhimurium, *S.* Tm) is a foodborne pathogen and one of the most prevalent non-typhoidal *Salmonella enterica* serovars affecting humans (11, 12). The infected phagocytes (mainly macrophages) migrate within the host body, helping the bacteria spread to other organs such as the spleen and liver. In these organs, the pathogenic bacteria proliferate preferentially (13). The phagocytes infected with *Salmonella* can also enter the bloodstream from the outer side of the intestinal base and circulate throughout the body.

This growing body of research highlights the critical role of microbial dysbiosis and specific pathogens in the development and progression of intestinal inflammation, offering potential avenues for therapeutic intervention. *Escherichia coli* Nissle 1917 (EcN, serotype O6:K5:H1) is a probiotic strain originally isolated from a soldier resistant to diarrhea during an outbreak (14). EcN is a Gram-negative probiotic that is sensitive to serum and does not produce intestinal toxins or cytotoxins associated with pathogenic *E. coli* strains (15, 16). EcN, widely used in Europe has been shown to establish sustained colonization in the gut and has been employed to treat or prevent various intestinal diseases, including acute enteritis, although the mechanisms underlying its protective effects remain unclear (17, 18). The *arcA* gene in EcN encodes arginine deiminase and plays an important role in the arginine decomposition pathway. The degradation of arginine via the arginine deiminase pathway supports anaerobic metabolism in many pathogen. This pathway consumes arginine to generate ammonia, ornithine, and carbon dioxide, and coupling the phosphorylation of ADP to ATP, and providing bacteria with energy in the absence of oxygen.

Studies on intestinal inflammation have shown changes in the composition and function of patients’ gut microbiota, as well as alterations in metabolite profiles (19, 20). Existing literature has demonstrated that by intervening in the metabolism of amino acids in the gut, the development of intestinal inflammation can be regulated (21). Additionally, during inflammation, local tissue metabolism changes, and the activation of white blood cells produces acidic substances, leading to a downward trend in blood pH (22, 23). In the inflammatory state, alkaline amino acids can bind to excess acids, acting as buffers, thereby participating in the regulation of the inflammatory response (24, 25). Therefore, we hypothesized that EcN may treat intestinal inflammation caused by *S.* Tm infection through the metabolic production of basic amino acids. Knockout of genes involved in basic amino acid catabolism significantly enhances the amino acid accumulation capacity of the strain. This allows for comparative analysis of differences in intervention effects between the mutant and wild-type EcN, providing experimental evidence for elucidating the anti-inflammatory mechanism mediated by amino acid metabolism.

In this study, we found that EcN regulates macrophage apoptosis via arginine metabolism, thereby mitigating *S.* Tm-induced intestinal inflammation. Mechanistic studies revealed that arginine can inhibit the activation of the mitochondrial apoptosis pathway by binding to the ribosomal protein S3 (RPS3) of macrophages. Our findings provide important insights into macrophage death caused by *S.* Tm infection, offering new targets and strategies for treating bacterial infection-induced intestinal inflammation.

## Materials and methods

### Animals

Six-week-old BALB/c female mice were purchased from Jiangsu Qinglongshan Biotechnology Co., Ltd. and housed under standard conditions. All animal procedures were approved by the Science and Technology Department of Jiangsu Province (Approval code: SYXK 2024-0001).

### Bacterial strains and cells

*Salmonella* Typhimurium SL1344 strain used in this study was available in our laboratory. *E.coli* Nissle 1917 strain, pTargetF plasmid, pCas plasmid and puc19-tlpa36-Wasabi plasmi were donated by Dr. Liming Song, Shenzhen Institute of Advanced Technology, Chinese Academy of Sciences.

RAW264.7 cells (ATCC TIB-71™) were maintained in DMEM supplemented with 10% heat-inactivated fetal bovine serum (Gibco) at 37 °C with 5% CO_2_.

### Adhesion and invasion inhibition assays

For the EcN treatment groups, EcN were grown in LB medium to an optical density at 600 nm (OD_600_) of approximately 1, washed by centrifugation, re-suspended in cell culture medium and adjusted by dilution to provide a multiplicity of infection (MOI) of 50∶1. RAW264.7 cells were first incubated with the respective *E. coli* strain for 1 hour at 37 °C. Cells were washed three times with PBS to remove non-adherent *E. coli*. *S.* Typhimurium was grown in LB medium to an OD_600_ of approximately 2, washed by centrifugation, re-suspended in cell culture medium and adjusted by dilution to provide a MOI of 100∶1. For the experiment of intervention with exogenous arginine, RAW264.7 cells were treated with (10、100 μM) or without arginine for 12 h. After discarding the supernatant, the cells were washed three times with PBS. Subsequently, the cells were infected with *S.* Typhimurium at an MOI of 50.

Adhesion assays were performed essentially as previously described (26) After *S.* Typhimurium infection at 37 °C, 5% CO_2_ for 1 h, the infected cell monolayers were washed with PBS three times. Cells were lysed in deionized water using 1% Triton X-100 (Sigma). Subsequently, the bacteria were counted using selective agar plates. Repeat three to five experiments for each measurement.

Bacterial invasion was assessed using a gentamicin protection assay, modified from previously described methods (27). After *S.* Typhimurium infection at 37 °C, 5% CO_2_ for 1 h, the infected cells were washed thrice with sterile PBS and incubated with DMED containing 100 μg/mL gentamicin for 1 h. Following incubation and PBS washing, cells were lysed using 1% Triton X100. The lysates underwent serial dilution, and bacterial invasion was quantified by plating on selective agar plates.

The number of extracellular bacteria was determined by aspirating the supernatant after *S.* Typhimurium infection at 37 °C, 5% CO_2_ for 2 h, followed by serial dilution plating on selective agar plates.

Adhesion rate = (number of adherent bacteria)/(number of infecting bacteria per well) × 100%; Invasion rate = (number of invaded bacteria)/(number of infecting bacteria per well) × 100%;Extracellular bacteria inhibition rate (Bacteriostatic rate) = (number of extracellular bacteria)/(number of infecting bacteria per well) × 100%.

### The optimal MOI

For the EcN treatment groups, RAW264.7 cells were pretreated with EcN for 1 hour first (MOI = 50) and then infected with *S.* Typhimurium (MOI = 100). Cells were collected for detection 2 hours later.

For the experiment of intervention with exogenous arginine, RAW264.7 cells were treated with or without arginine (10、100 μM) for 12 h, then infected with *S.* Typhimurium at an MOI of 50 for 2 h.

### Non-targeted metabolomic analysis

Harvest fresh colon tissue, and quickly wash the surface of the tissue to remove stains with 1×PBS prepared using pre-cooled enzyme-free water. Blot dry the liquid on the surface. Neatly arrange the tissue and promptly place it into a pre-cooled, enzyme-free, screw-threaded cryotube with a screw cap that can withstand -192 °C and has the number already written on it. Flash-freeze the tissue in liquid nitrogen for 3 to 4 hours, and then store it at -80 °C. Employ the LC-MS/MS technique for non-targeted metabolomics analysis.

### Identification of potential arginine-binding proteins from RAW264.7 cells by MS

Proteins in macrophages lysates were collected and diluted in 5 × SDS‐PAGE sample buffer. Protein samples were loaded on 8% SDS‐PAGE gels. The proteins in the gel strips are enzymatically digested and desalted. Subsequently, mass spectrometry parameters are set for analysis, and the obtained data are compared with a selected database.Finally, functional enrichment analysis is carried out using the Gene Ontology (GO) and Kyoto Encyclopedia of Genes and Genomes (KEGG) databases.

### Data collection and analysis

CTD query (http://ctdbase.org/search/) was used to retrieve and analyze all data sets. Overlapping differentially expressed genes among patients with *Salmonella* Typhimurium infection, Ulcerative Coliti and Crohn’s Disease were subjected to KEGG pathway enrichment analysis using the clusterProfiler package and the org.Hs.eg.db database. The enrichKEGG method was applied with a pvalueCutoff of 0.05 and a qvalueCutoff of 0.05, yielding 189 significantly enriched pathways.

### Bacterial culture and constructs

The construction of *S.* Typhimurium carrying the pUC19-tlpa36-Wasabi plasmid was accomplished by transforming chemically competent bacterial cells with a pUC19-based plasmid harboring the *tlpa36* gene and the Wasabi fluorescent protein gene under the control of the lac promoter. Transformants were selected on ampicillin-containing LB agar plates and validated by colony PCR and fluorescence microscopy. The resulting strain expresses Wasabi fluorescent protein, enabling real-time visualization and tracking of *tlpa36* gene expression and bacterial behavior *in vitro* and *in vivo*.

The knockout of *arcA* was accomplished using the pTargetF and pCas plasmids. In brief, Select the target site ctaacacctgacggtcacgg to construct the sgRNA plasmid, pTargetF-sg-*arcA*. Using the primer F1: tgctttgaaaacccagcc, R1: gaattaggccgcggac, and the Nissle1917 genome as the template, the left homologous arm is obtained by PCR. Using the primer F2: gtccgcggcctaattccgctgcgtagttgtcc, R2: ctctcagtcagatcacagttc, and the Nissle1917 genome as the template, the right homologous arm is obtained by PCR. The homologous recombination fragment Donor is obtained by overlap extension PCR of the left and right homologous arms. Then, the plasmid pCas is electroporated into Nissle1917. Single colonies are picked, and competent cells are prepared by adding 10 mM of arabinose. The pTargetF-sg-*arcA* and Donor are electroporated into the competent cells of pCas-Nissle1917. Single colonies are picked, and the knockout is verified by PCR (28).

To construct the complementation plasmid pUC19-LacI-*arcA*, the Beyotime seamless cloning kit was employed for fragment assembly. Briefly, the plasmid pUC19 was used as a template and subjected to double digestion with PciI and EcoO109I. The linearized vector was gel-purified. Using EcN genomic DNA as a template, two insert fragments were amplified via PCR with the following primer pairs: LacI-F (CCTGCGTTATCCCCTGATTCGACACCATCGAATGGCG) and LacI-R (TCTCCTTCTTAAAGTTAAACAAATTCCACAATTCACCACCCTGAATTGAC) for the LacI fragment, and arcA-F (TTTGTTTAACTTTAAGAAGGAGAaataatgatggaaaagcattatgtt) and arcA-R (AAAATAGGCGTATCACGAGGttagataccgtcacgatgc) for the *arcA* fragment. The PCR products were separated by agarose gel electrophoresis and purified accordingly. The linearized vector and the two insert fragments were then ligated using the seamless cloning kit. The resulting ligation product was transformed into DH5α competent cells, and positive clones were selected for sequencing verification.

### Disease activity index

Mice were monitored weekly for body weight changes and stool consistency.

### qPCR experiments

Total RNA was extracted using TRIzol Reagent (Vazyme) and reverse-transcribed to cDNA using HiScript^®^ II Reverse Transcriptase (Vazyme). RT-PCR was performed using ChamQ Universal SYBR qPCR Master Mix.

### H&E staining and histological assessment

Colon tissues were stained with H&E according to standard protocols.

### Immunofluorescence staining

Paraffin sections of colon tissue were stained with F4/80, cleaved Caspase-3,occludin and ZO-1 antibodies (UpingBio, China), followed by Alexa Fluor^®^ 647 secondary antibody. Immunohistochemical (IHC) staining was performed as described previously (29). Images were acquired using a Nikon confocal microscope.

### Cell membrane integrity detection

Propidium iodide (PI) is a small fluorescent molecule that binds to DNA but cannot passively traverse into cells with an intact plasma membrane. PI uptake versus exclusion can be used to discriminate dead cells, in which plasma membranes become permeable regardless of the mechanism of death, from live cells with intact membranes.

### GeneMANIA database

The GeneMANIA database (http://genemania.org/) (30) is designed to predict the functions of genes of interest. It indexes 2,830 association networks containing 660,554,667 interactions mapped to 166,691 genes from nine organisms. This database enables users to predict gene-gene functional interaction networks from a provided gene list. In the present study, the GeneMANIA database was used to predict interactions among RPS3, SRSF3, BCL2, BAX, and cleaved CASPASE3.

### HitPredict database

HitPredict (http://www.hitpredict.org/) (31) integrates protein-protein interactions derived from high-throughput or small-scale experiments in the IntAct, BioGRID, HPRD, MINT, and DIP databases. In this study, by searching the HitPredict database for the human gene symbol RPS3, the associated proteins that interact with RPS3 were identified.

### Mass spectrometry analysis of amino acids

An Ultimate 3000 ultra-high-performance liquid system with Orbitrap-Exploris 120 Mass Spectrometer (Thermo Fisher Scientific, San Jose, CA, USA) was performed for sample separation and detection. Lysine (MW 146.19), arginine (MW 174.20), and histidine (MW 155.15) were analyzed using the following conditions: flow rate, 1.0 ml/min; detection wavelength, 254 nm; column temperature, 30 °C. Mass spectrometry was performed using a Q3 scan with a mass range of 50.00–300.00 m/z. The elution gradient was 10% acetonitrile (+1‰ FA) and 90% water (+1‰ FA), with an elution time of 10 min.

### PI nuclear staining

Untreated and tyloxapol-treated cells (2×10^5) were incubated and harvested. The cells were fixed with 1% paraformaldehyde for 60 min at room temperature and then washed three times with 0.1% Tween 20 in PBS. The cells were then incubated with PI staining solution (40 μg/ml PI, 100 μg/ml RNase A) for 30 min in the dark. After washing five times with PBS, the cells were viewed under a fluorescent microscope.

### DNA fragmentation assays

Cells (1×10^6) were washed twice with PBS, suspended in 500 μL of lysis buffer (20 mM Tris, 10 mM EDTA, 0.2% Triton X-100; pH 8.0), and incubated on ice for 10 min. After centrifugation at 1200 rpm for 10 min, the cell lysate was incubated with proteinase K (final concentration = 200 μg/ml) to digest proteins at 50 °C for 8 h, followed by incubation with RNase A (final concentration = 100 μg/ml) to digest RNA at 37 °C for 6 h. DNA was extracted twice, first using saturated phenol solution and then using chloroform plus isoamyl alcohol. After centrifugation at 12,000 rpm for 10 min, glycogen (final concentration = 20 μg/ml) and an equal volume of isopropanol were added to the upper aqueous layer. The mixture was stored at −20 °C for 24 h. The extracted DNA was dissolved in TE buffer solution (10 mM Tris, 1 mM EDTA; pH 8.0) and subjected to 2% agarose gel electrophoresis at 100 V for 30 min.

### ROS detection

ROS levels were assessed using a Reactive Oxygen Species Assay Kit. After harvesting and rinsing with ice-cold PBS, cells were stained with DCFH-DA (10 μM) at room temperature for 30 min in the dark. Samples were analyzed using a FACS Canto™ II cytometer, and data were processed using FlowJo^®^ software.

### Western blotting

Cell samples were subjected to SDS-PAGE, and proteins were transferred to polyvinylidene difluoride (PVDF) membranes (Millipore). After blocking with 5% skim milk or BSA in TBST, the membranes were incubated overnight at 4 °C with primary antibodies, including anti-BCL2, anti-BAX, anti-cleaved CASPASE3, anti-RPS3, anti-SRSF3, anti-GAPDH (Biodragon, China), and anti-actin antibodies. These antibodies were purchased from Santa Cruz. After incubation with rabbit-conjugated secondary antibody (Cell Signaling Technology) at room temperature for 2 h, the membranes were soaked in ECL solution, and images were captured using a luminescence imaging system.

### Statistical analysis

Data from independent experiments are presented as means ± standard deviations. Comparisons between two datasets were analyzed using an unpaired Student’s t-test. For three or more datasets, one-way analysis of variance with Tukey’s multiple comparisons post-test was used. Statistical analysis was performed using SPSS (version 18.0) software, and data processing was conducted using Prism (version 8.0). A P value of <0.05 or <0.01 was considered statistically significant.

### Confocal Imaging

To observe internalization, cell nuclei were labeled with DAPI, and intracellular mitochondria were labeled with MitoTracker Red CMXRos. Macrophages were treated with *Salmonella* Typhimurium for 2 h, washed, and imaged using confocal microscopy. To assess mitochondrial localization, macrophages were infected with *Salmonella* Typhimurium for 2 h, fixed with 4% paraformaldehyde for 30 min at room temperature, permeabilized with 0.1% Triton X-100 for 30 min, blocked with 1% bovine serum albumin for 30 min, and stained with MitoTracker Red CMXRos at 37 °C for 30 min. All samples were imaged using a Carl Zeiss LSM 800 confocal microscope, and images were analyzed using ZEN Black software. Data are from two independent experiments, each performed in triplicate.

### Cell culture and transfection

RAW264.7 cells were purchased from ATCC and cultured in DMEM containing 10% FBS without antibiotics (penicillin and streptomycin). All cell lines used in the laboratory were authenticated and tested for mycoplasma contamination. Cells were transfected with indicated plasmids using Lipofectamine 2000 (Invitrogen), while siRNA transfections were performed using Lipofectamine RNAiMAX, according to the manufacturer’s instructions. The siRNA used was as follows: siRPS3 5′-GCTGCCTATGATGTCTCGTTT-3′.

### Dosage information

For the EcN intervention model, each mouse was administered 1×10^6 CFU of EcN (200 µL suspension in PBS) or treated with 200 µL sterile PBS (control) by oral gavage every other day. After pretreatment for 1 week, mice were infected with 1×10^8^ CFU of *Salmonella* Typhimurium strain SL1344, with oral gavaged performed every two days. During the arginine intervention, dissolve arginine in PBS to final concentrations of 5 and 50 mg/mL, respectively. Intraperitoneally inject 200 μL of the solution into each mouse. After pretreatment for 1 week, mice were infected with 1×10^7^ CFU of *Salmonella* Typhimurium strain SL1344, with oral gavaged performed every two days. Body weights were measured following each oral gavage administration. For the continuous infection model, mice were sacrificed 48 hours post the sixth infection. Colon tissue samples from the intestinal tracts were collected for analysis.

### *Salmonella* burden in colon tissue

For theinitial colonization detection, the mice were first pre-treated with the above-mentioned EcN/arginine, and then a single dose of *S.* Tm was administered to them orally. 24、48 hours post-infection, colon contents were collected, resuspended in sterile PBS, then bacterial load for *S*. Typhimurium strains was determined by plating serial 10-fold dilutions on selective agar plates (SenBeiJiabio, Nanjing,China) (32).

For the detection of *S.* Tm colonization after continuous infection, at 24 and 48 hours after the sixth infection, colon tissues were harvested from each mouse, washed with sterile PBS to remove intestinal contents and luminal bacteria, and serially diluted 1:10 in LB. Aliquots (100 µL) were plated on *Salmonella*-selective agar medium (SenBeiJiabio, Nanjing,China). Plates were incubated overnight at 37 °C, and bacterial colonies were counted.

### Growth curve

*S.* Tm and EcN were prepared by inoculating 100 µL of overnight-cultured frozen stock into 5 mL of fresh LB medium and incubating statically at 37 °C (5% CO_2_). At 0, 2, 4, 6, 8, 10 and 12 h, culture was sampled and diluted 10-fold serially using LB. The diluted culture was spread onto LB agar plates and incubated at 37 °C for 18–24 h. The colony-forming units (CFUs) were counted, and the bacterial concentration in the original sample was calculated. Triplicate absorbance readings were compared with time to generate bacterial growth curves.

### TEM

Bacteria were cultured as described above. Cellular collectives were fixed with 2.5% glutaraldehyde for 24 h, embedded in 2% agarose, and processed by the Electron Microscopy Research Service of China Pharmaceutical University. Cell morphology was observed using Image-Pro Plus (version 6.0).

### Mitochondrial transmembrane potential assay

The ΔΨm was evaluated using a JC-1 kit (Solarbio, China) as previously described (33). JC-1 is a fluorescent probe indicating mitochondrial membrane potential loss. In normal cells, JC-1 accumulates in the mitochondrial matrix, forming a polymer that exhibits red fluorescence; in cells with low ΔΨm, JC-1 exists as a monomer and yields green fluorescence. Cells were incubated with 5 µM JC-1 solution in the dark at 37°C for 20 min after treatment with indicated stimulators. The cells were then washed with buffer and imaged under a fluorescence microscope.

### Annexin V/propidium iodide staining and flow cytometry

The RAW264.7 cell line (1×10^6^ cells/well) was grown on six-well plates, and apoptotic rates were evaluated using the Annexin V/PI method. The cell culture medium was aspirated and discarded, and cells were rinsed twice with PBS (500 µL). Trypsin (500 µL) was added and incubated for 1–2 min, followed by the addition of complete medium (500 µL) to stop digestion. Cells were transferred to a 1.5-mL centrifuge tube and centrifuged at 1000 rpm for 5 min. The cell pellet was resuspended in 500 µL binding buffer. Cells in the PI single-staining group were heated in a water bath at 53 °C for 5 min, while those in other treatment groups were incubated at room temperature for 15–30 min. After centrifugation, the cell pellet was resuspended again in 500 µL binding buffer. Samples were analyzed using a FACS Canto™ II cytometer (BD Biosciences) at 535 nm (PI) and 488 nm (Annexin V). Data were analyzed using FlowJo^®^ software (Tree Star, OR, USA).

### Co-IP

Cell lysates were prepared using IP lysis buffer (P0013, Beyotime) supplemented with a protease inhibitor cocktail. Lysates were incubated with Dynabeads™ Protein A magnetic beads (10001D, Thermo Fisher Scientific) for 2 hours at 4 °C with rotation. The pre-cleared lysates were then immunoprecipitated overnight at 4 °C with either anti-RPS3 antibody or control IgG (1:50, ab172730, Abcam), followed by incubation with 20 μL of pre-washed magnetic beads for 2 hours. The bead-bound immunocomplexes were washed three times with cold IP buffer, and the co-precipitated proteins were eluted for subsequent Western blotting analysis.

### DAPI staining

Chromatin shrinkage in RAW264.7 cells was evaluated by DAPI staining. RAW264.7 cells were inoculated in six-well plates, washed three times with PBS after *Salmonella* Typhimurium infection, and fixed with 4% paraformaldehyde at room temperature for 15 min. Cells were stained with DAPI solution at room temperature in the dark for 10 min, washed with PBS, and photographed under an Olympus IX71 inverted fluorescence microscope.

## Results

### Differences of arginine metabolism in *S.* Tm infection

**Figure 1:**
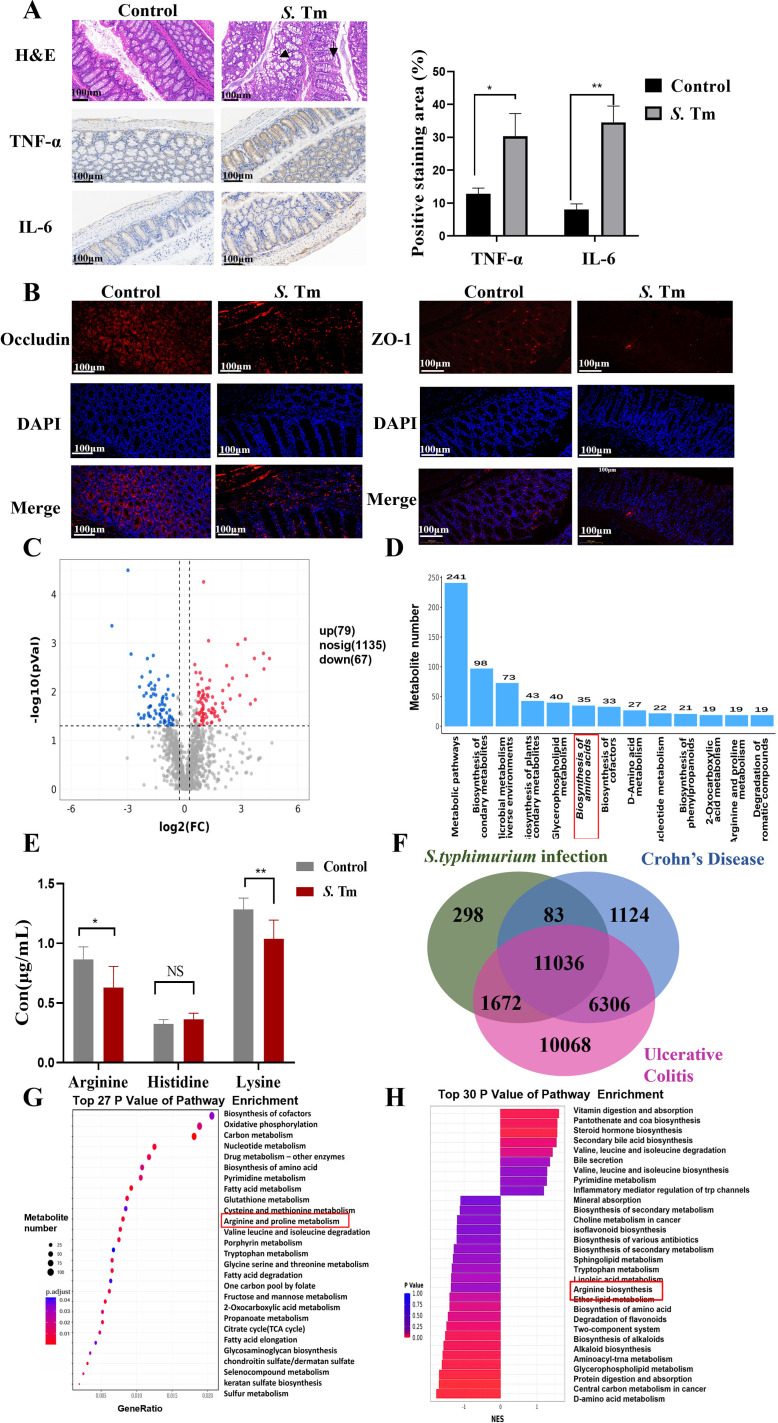
Differences of arginine metabolism in *S*. Tm infection **(A)** Representative images of haematoxylin and eosin (H&E) staining and immunohistochemistry (IHC) staining of TNF-α and IL-6 in mice colon tissue (*n* = 5 mice per group, 1×10^6 CFU/mouse/d, 14d). **(B)** Immunofluorescence (IF) comparison of protein expression of Occludin and ZO-1 in mice colon tissue (*n* = 5 mice per group). **(C)** Volcanic maps identify differential metabolites by non-targeted metabolomics analysis. **(D)** Metabolite number TOP20 pathway. **(E)** Analysis of serum arginine, histidine, and lysine by mass spectrometry (*n* = 5 mice per group). **(F-G)** Venn diagram of overlapping differentially expressed proteins in patients with *Salmonella* infection, Crohn’s disease, and ulcerative colitis **(F)** and kegg enrichment analysis of metabolism-related differential genes **(G, H)** Differential metabolite GSEA enrichment analysis. The data are reported as the mean ± SD. NS, not significant differences; *p < 0.05; **p < 0.01; ***p < 0.001.

To explore the metabolic mechanism underlying intestinal inflammation caused by *S*.Tm infection, this section first analyzed the impact of *S*.Tm infection on the metabolic profile of the mice’s intestines. Oral gavage of mice with 20 mg streptomycin, followed by a 20-hour treatment and 4-hour fasting, was performed prior to *Salmonella* infection experiments (34). Histological analysis of colon tissue was conducted to assess the impact of *S.* Tm infection on intestinal homeostasis. Haematoxylin and eosin (H&E) staining revealed disrupted colonic epithelial cell arrangement and infiltration of inflammatory cells. Further immunohistochemical (IHC) staining demonstrated that *S.* Tm infection increased the expression of tumor necrosis factor-alpha (TNF-α) and interleukin- 6 (IL-6) (**Figure 1A**). Immunofluorescence (IF) analysis showed reduced expression of tight junction proteins ZO-1 and Occludin, which are critical for maintaining intestinal barrier integrity (**Figure 1B**).

Untargeted metabolomics was employed to analyze metabolites in mouse colon tissue, investigating the metabolic alterations induced by *S.* Tm infection. Compared to the control group, the infection model group exhibited 146 differentially expressed metabolites (79 upregulated, 67 downregulated) (**Figure 1C**). KEGG pathway analysis of these metabolites revealed significant changes in multiple metabolic pathways, particularly amino acid metabolism (**Figure 1D**). Given the potential anti-inflammatory role of basic amino acids, we quantified the levels of arginine, histidine, and lysine in blood samples using HPLC-MS (**Figure 1E**). The results showed a significant reduction in arginine and lysine in *S.* Tm-infected mice.

Analysis of the CTD (Comparative Toxicogenomics Database) revealed overlapping differentially expressed genes among Crohn’s Disease patients, Ulcerative Colitis patients, and *Salmonella* Typhimurium-infected individuals, suggesting that *Salmonella* infection may be a key trigger for intestinal inflammation and could contribute to intestinal inflammation progression (**Figure 1F**). KEGG enrichment analysis (enrichKEGG) of these overlapping genes identified 189 significantly enriched pathways, with “Arginine and proline metabolism” being among the most prominent metabolic pathways (**Figure 1G**). Additionally, Gene Set Enrichment Analysis (GSEA) of colon tissue metabolites from infected mice showed significant enrichment in arginine metabolic pathways (**Figure 1H**). These findings suggest that arginine plays a pivotal role in maintaining physiological homeostasis and enhancing host defense against infection in mice.

### Arginine mediates EcN to inhibit macrophage apoptosis induced by *S.* Tm

**Figure 2:**
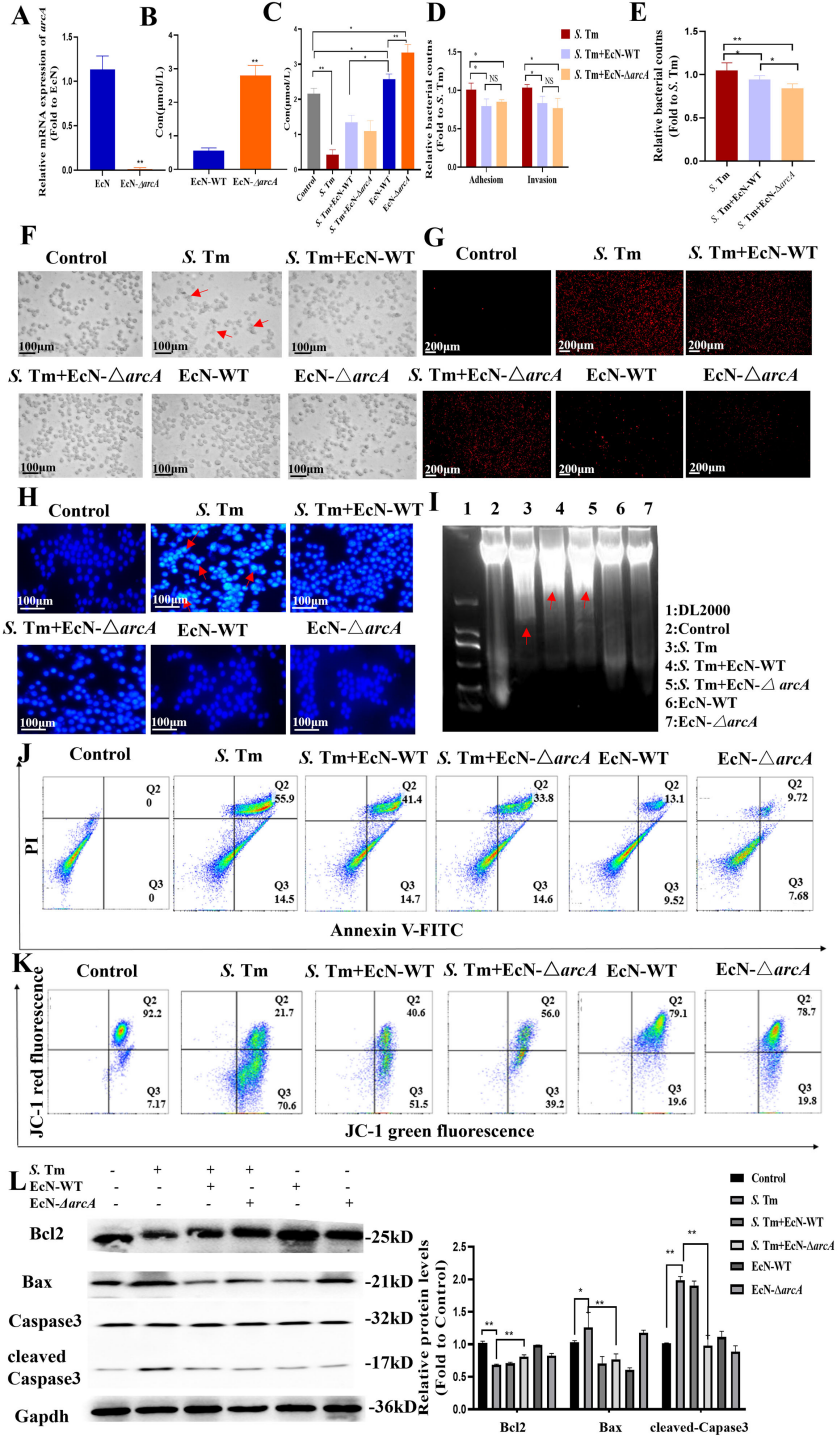
Arginine mediates *E coli* Nissle1917 to inhibit macrophage apoptosis induced by *S*. Tm **(A)** Validation of *arcA* knockout efficiency by reverse transcription-quantitative RT–qPCR (*n* = 5 independent experiments). **(B,C)** Arginine production in bacteria **(B)** and cell **(C)** was detected by colorimetric method (*n* = 5 independent experiments). **(D)** Adhesion and invasion of *S.* Tm in RAW264.7 cells (*n* = 5 independent experiments). **(E)** Bacteriostatic rates of RAW264.7 cells against *S.* Tm (*n* = 5 independent experiments). **(F)**Morphology of macrophages in different groups (*n* = 5 independent experiments). **(G)** The permeability of cell membrane was detected by PI staining (*n* = 5 independent experiments). **(H)** DAPI staining for detection of chromatin condensation (*n* = 5 independent experiments).**(I)** Agarose gel electrophoresis demonstrating the DNA fragmentation (*n* = 5 independent experiments). **(J)** Annexin V FITC/PI staining was used to detect apoptosis (*n* = 5 independent experiments). **(K)** JC-1 assay for detection of mitochondrial membrane potential changes (*n* = 5 independent experiments).**(L)** Western blot of pro and anti-apoptotic proteins, Gapdh was used as housekeeping control (*n* = 5 independent experiments). The data are reported as the mean ± SD. NS, not significant differences; *p < 0.05; **p < 0.01.

Based on the protective effect of EcN in intestinal inflammation and the crucial function of arginine in maintaining intestinal homeostasis, we hypothesized that EcN might exert its therapeutic effect on intestinal inflammation by metabolically producing arginine. We designed an *arcA* gene knockout strain through homologous recombination, and the obtained EcN-Δ*arcA* strain was verified by PCR (**Figure 2A**). To clearly determine the effect of *arcA* on the arginine metabolism of EcN, we detected the arginine production of EcN before *arcA* knockout using an arginine concentration detection kit. The results indicated a strong correlation between *arcA* gene and arginine metabolism (**Figure 2B**). Additionally, measurement of intracellular arginine concentration in RAW264.7 cells revealed that treatment with EcN, particularly the EcN-Δ*arcA* strain, significantly increased intracellular arginine concentrations in macrophages. This observation suggests that arginine in the EcN (EcN-WT and EcN-Δ*arcA*) strains is efficiently transported into macrophages, thereby establishing a favorable metabolic microenvironment for the host to combat *S*. Tm infection (**Figure 2C**).

To test whether arginine accumulation is essential for the subsequent colonization of S. Tm by EcN, we compared adhesion and invasion rates of Salmonella between EcN-WT and another *arcA* gene-negative EcN strains. As shown in **Figure 2D**, compared with *S.* Tm infection alone, EcN pretreatment reduced the adhesion rate of *S.* Tm. Additionally, no correlation was observed between arginine accumulation and the inhibitory effect of EcN on *S.* Tm adhension. Pretreatment with EcN can reduce *S.* Tm invasion, and this effect is not significantly correlated with arginine accumulation (**Figure 2D**), which means that while EcN significantly inhibits the adhesion and invasion of *S.* Tm into RAW264.7 cells, this inhibitory effect is not potentiated by arginine accumulation. We further examined the impact of EcN or arginine accumulation on the number of extracellular bacteria. The results showed that EcN-WT intervention led to a significant reduction in extracellular bacterial counts, and this beneficial effect was more pronounced following pretreatment with EcN-Δ*arcA* (**Figure 2E**). These findings indicate that the regulation of macrophage extracellular antibacterial function by EcN is dependent on the negative regulation of the *arcA* gene, whereas arginine accumulation may further enhance macrophage-mediated clearance of extracellular bacteria.

To delineate arginine’s role in post-invasion pathogenesis, we systematically evaluated whether intracellular arginine accumulation modulates EcN’s capacity to mitigate *S.* Tm-triggered macrophage damage. Light microscopy showed that *Salmonella* infection caused significant morphological changes in RAW cells, with membrane disintegration and fragmentation at the cell edges, indicating damaged cellula integrity. However, EcN pretreatment preserved macrophage membrane integrity with distinct peripheral margins and reduced cellular shrinkage, suggesting a protective role in maintaining cellular homeostasis (**Figure 2F**). The damage of macrophages was evaluated by propidium iodide (PI) staining. The results showed that the PI staining intensity and the number of macrophages in the EcN-Δ*arcA* treatment group were significantly lower than those in the EcN-WT group (**Figure 2G**), indicating better cell membrane integrity.

We further detected the chromatin condensation through 4’,6-diamidino-2-phenylindole (DAPI) staining. The results showed that the EcN-WT group could significantly reduce the nuclear condensation in cells caused by *S.* Tm infection. Compared with the EcN-WT group, the nuclear condensation in the EcN-Δ*arcA* treatment group was significantly lower (**Figure 2H**). The DNA degradation in cells was detected using a DNA extraction kit. The results showed that both the EcN-WT group and the EcN-Δ*arcA* group were able to reduce to some extent the intracellular DNA fragmentation phenomenon caused by *S*. Tm infection (**Figure 2I**).

Considering that the role of EcN in the apoptosis of macrophages induced by *S.* Tm has not been reported, we further detected the effect of arginine accumulation on the process of macrophage apoptosis after EcN inhibited *S*. Tm infection by flow cytometry. The results showed that the survival rate of macrophages was significantly increased under the condition of arginine accumulation (**Figure 2J**).

The mitochondrial membrane potential (ΔΨm) reflects the functional metabolic state of mitochondria, which is also a key event in cell apoptosis. The results of detecting the mitochondrial membrane potential by JC-1 showed that, compared with the EcN-WT group, the mitochondrial membrane potential in the EcN-Δ*arcA* treatment group was significantly increased (**Figure 2K**). According to the above results, we detected the key proteins in the mitochondrial apoptosis pathway. The results showed that EcN under the condition of arginine accumulation inhibited the downregulation of B-cell lymphoma-2 (BCL2) in macrophages caused by *S*. Tm infection, and at the same time, hindered the enhancement of Bcl-2-associated X protein (BAX) expression and the activation of CAPASE3 (**Figure 2L**).

**Figure 3:**
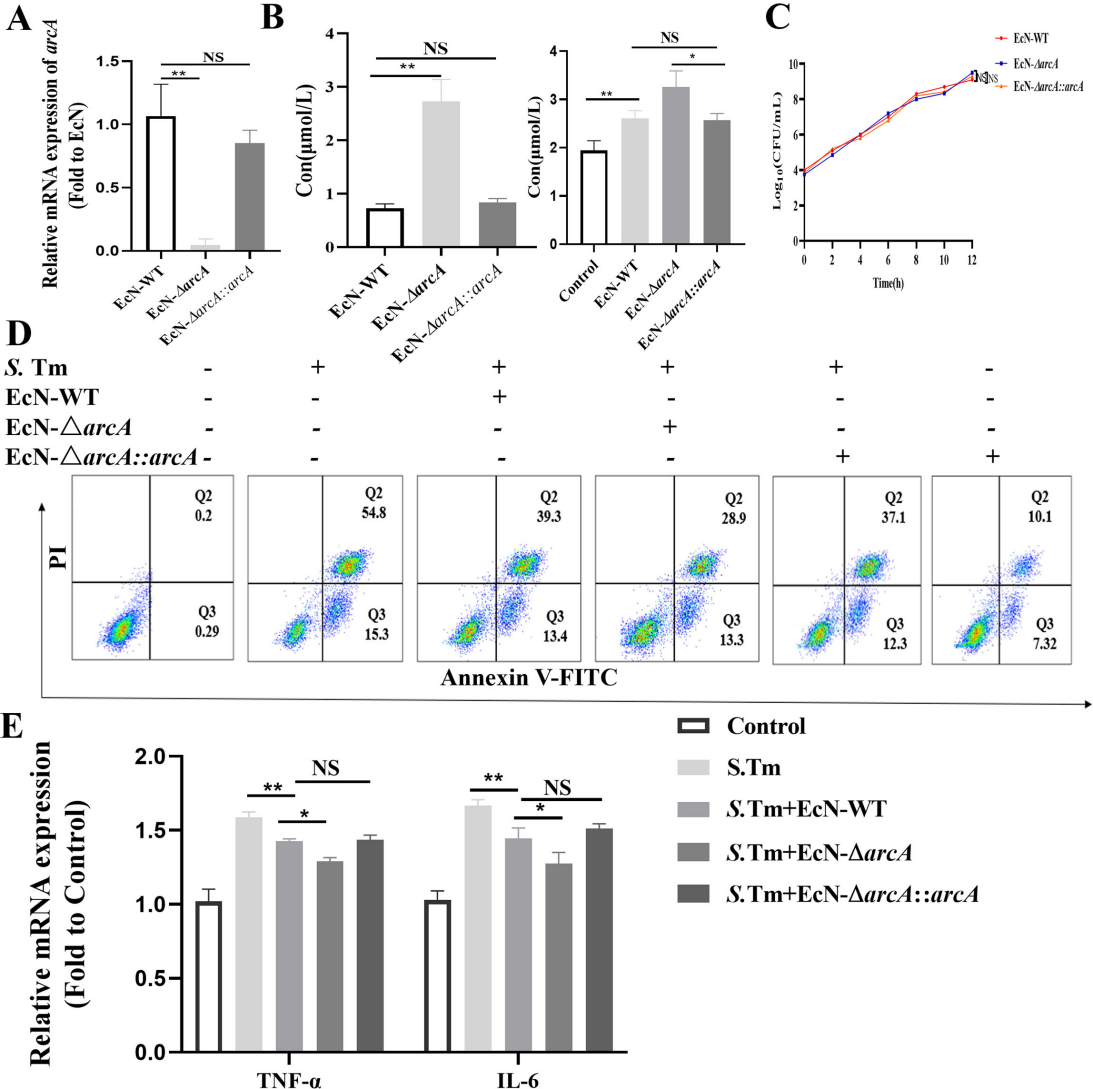
Effects of *arcA* knockout on the inhibition of Salmonella-induced macrophage apoptosis by EcN. **(A)** Validation of *arcA* knockout efficiency by reverse transcription-quantitative real-time PCR (*n* = 5 independent experiments). **(B)** Arginine production in bacteria (left) and cell (right) was detected by colorimetric method (*n* = 5 independent experiments). **(C)** Bacteria growth curves. **(D)** Relative mRNA expression levels of TNF-α and IL-6 (*n* = 5 independent experiments). **(E)** Representative flow cytometry analysis of macrophage apoptosis (*n* = 5 independent experiments). The data are reported as the mean ± SD. NS, not significant differences; *p < 0.05; **p < 0.01.

To unequivocally determine the impact of *arcA* on arginine levels, we further engineered the *arcA*-complemented strain EcN-Δ*arcA*::*arcA*, and the engineered strain was validated as effective by PCR (**Figure 3A**). Further detection revealed that compared with EcN-Δ*arcA*, both the arginine production capacity of the EcN-Δ*arcA*::*arcA* strain and the intracellular arginine level in pretreated cells were significantly reduced, indicating a strong correlation between *arcA* and the arginine metabolic level of the bacteria (**Figure 3B**). Subsequently, we detected that *arcA* knockout did not affect the growth of bacteria (**Figure 3C**). To investigate the role of *arcA* in the resistance of EcN to S. Tm infection, RAW264.7 cells were pretreated with three strains (EcN-WT, EcN-Δ*arcA*, and EcN-Δ*arcA*::*arcA*) respectively, followed by challenge with *S.* Tm respectively. Flow cytometry analysis revealed that compared with the EcN-Δ*arcA* strain, the ability of cells pretreated with EcN-Δ*arcA*::*arcA* to inhibit *S.* Tm infection-induced apoptosis was significantly reduced (**Figure 3D**). *Salmonella* infection typically induces a significant upregulation of inflammatory cytokine release in host cells (35, 36). Concomitantly, we investigated the regulatory effect of EcN preconditioning on the release level of tumor necrosis factor alpha (TNF-α)and interleukin-6 (IL-6) in host cells induced by *Salmonella* infection. The results showed that after *arcA* complementation, the ability of EcN-Δ*arcA* to inhibit the increase in TNF-α and IL-6 transcription level was significantly impaired, indicating that *arcA* plays a crucial role in the regulation of the homeostasis of cellular health by EcN (**Figure 3E**). Collectively, these results underscore the potential role of arginine in EcN as a therapeutic agent against *S*. Tm infection, laying the foundation for future clinical studies.

### Arginine mediates EcN’s inhibition of *S*. Tm-induced apoptosis in murine intestinal macrophages

**Figure 4:**
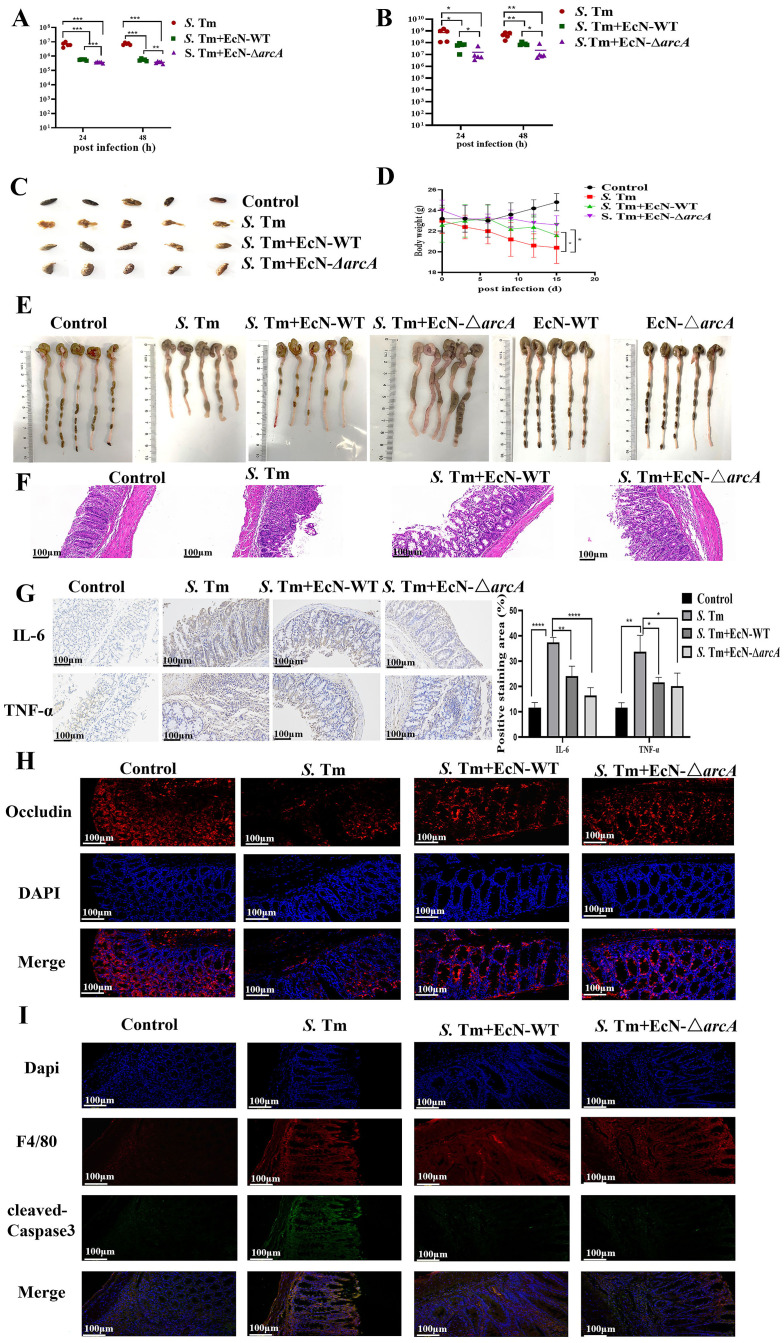
EcN improved chronic colitis conditions in the S. Tm-induced mouse model **(A)** Colonic bacterial load among different groups following primary *S.* Tm infection (*n* = 5 mice per group). **(B)** Colonic bacterial load among different groups following consecutive *S.* Tm infection (*n* = 5 mice per group). **(C)** Representative fecal samples from each group (*n* = 5 mice per group). **(D)** Body weight changes among different groups (*n* = 5 mice per group). **(E)** Colon samples and colon lengths measured from each group (*n* = 5 mice per group). **(F)** Representative H&E staining of colon sections from different treatment groups (*n* = 5 mice per group). **(G)** IHC staining was performed to detect IL-6 and TNF-α expression in the indicated colon samples (*n* = 5 mice per group). **(H)** IF staining comparison of Occludin expression in colon tissue of mice in different treatment groups (*n* = 5 mice per group). **(I)** The IF images of colon tissues from each group stained with F4/80 (red), cleaved-Caspase3 (green), and nuclear (blue) (*n* = 5 mice per group). The data are reported as the mean ± SD. NS, not significant differences; *p < 0.05; **p < 0.01; ***p < 0.001; ****p < 0.0001.

Next, we investigated the role of arginine in the treatment of mice challenged by *S*. Tm with EcN. Mice were then infected with *S*. Tm alone or pre-administered with EcN wild-type or mutants. Colonization in the colon content was then determined at 24、48 hours after the primary infection. The results showed that mice pretreated with EcN exhibited significantly reduced colonization levels of *S.* Tm strains in the intestinal tract compared to the untreated control group, and there was a significant difference in the colonization of *S.* Tm between the mice pretreated with EcN-WT and EcN-Δ*arcA* strains (**Figure 4A**). Moreover, under the condition of continuous infection with *S.* Tm, compared with EcN-WT, the administration of EcN-*ΔarcA* significantly reduced intestinal colonization with *S*. Tm (**Figure 4B**). These results indicate that arginine accumulation is essential for the beneficial effect of EcN pretreatment in reducing *S.* Tm colonization in mice.

We next analyzed whether the probiotic strain also ameliorated the intestinal inflammation caused by *S.* Tm in continuous infection model. Observation and analysis of the fecal characteristics of the mice revealed that, in contrast to the normal, dry feces of the control group mice, the mice in the model group exhibited typical diarrhea-like loose stools. In the EcN treatment group, however, the fecal characteristics of the mice were significantly improved, presenting a moist and soft state (**Figure 4C**). The EcN-WT group and the EcN-Δ*arcA* group both had a similar degree of inhibiting the weight loss in the model group mice (**Figure 4D**). Further dissection of the mouse colon showed that the overall morphological appearance of the mouse colon was restored (**Figure 4E**). H&E staining demonstrated that the mice in the EcN-Δ*arcA* treatment group had less infiltration of lymphocytes and granulocytes, as well as a small amount of intestinal epithelial cell shedding (**Figure 4F**).

Subsequently, IHC was employed to measure the levels of the apoptosis markers TNFα and IL-6 in intestinal inflammation. The results demonstrated that the intestinal inflammation in mice caused by *S*. Tm under the intervention of EcN-Δ*arcA* was weaker than that under the treatment of EcN-WT (**Figure 4G**). Immunofluorescence assay results indicated that, compared with EcN-WT, EcN-Δ*arcA* could more effectively inhibit the downregulation of the expression of the Occludin protein in the colon induced by *S*. Tm infection, which was in line with our expected results (**Figure 4H**).

Consistent with the *in vitro* data, the expression of cleaved- Caspase3 in macrophages in the EcN-Δ*arcA* treatment group was lower, as evidenced by the immunofluorescence co-localization of macrophages and cleaved-Caspase3 in the mouse colon (**Figure 4I**). Collectively, these findings suggest that arginine may play a crucial role in the ability of EcN to modulate host defense against infection.

### Arginine inhibits *S*. Tm-induced macrophage apoptosis

**Figure 5:**
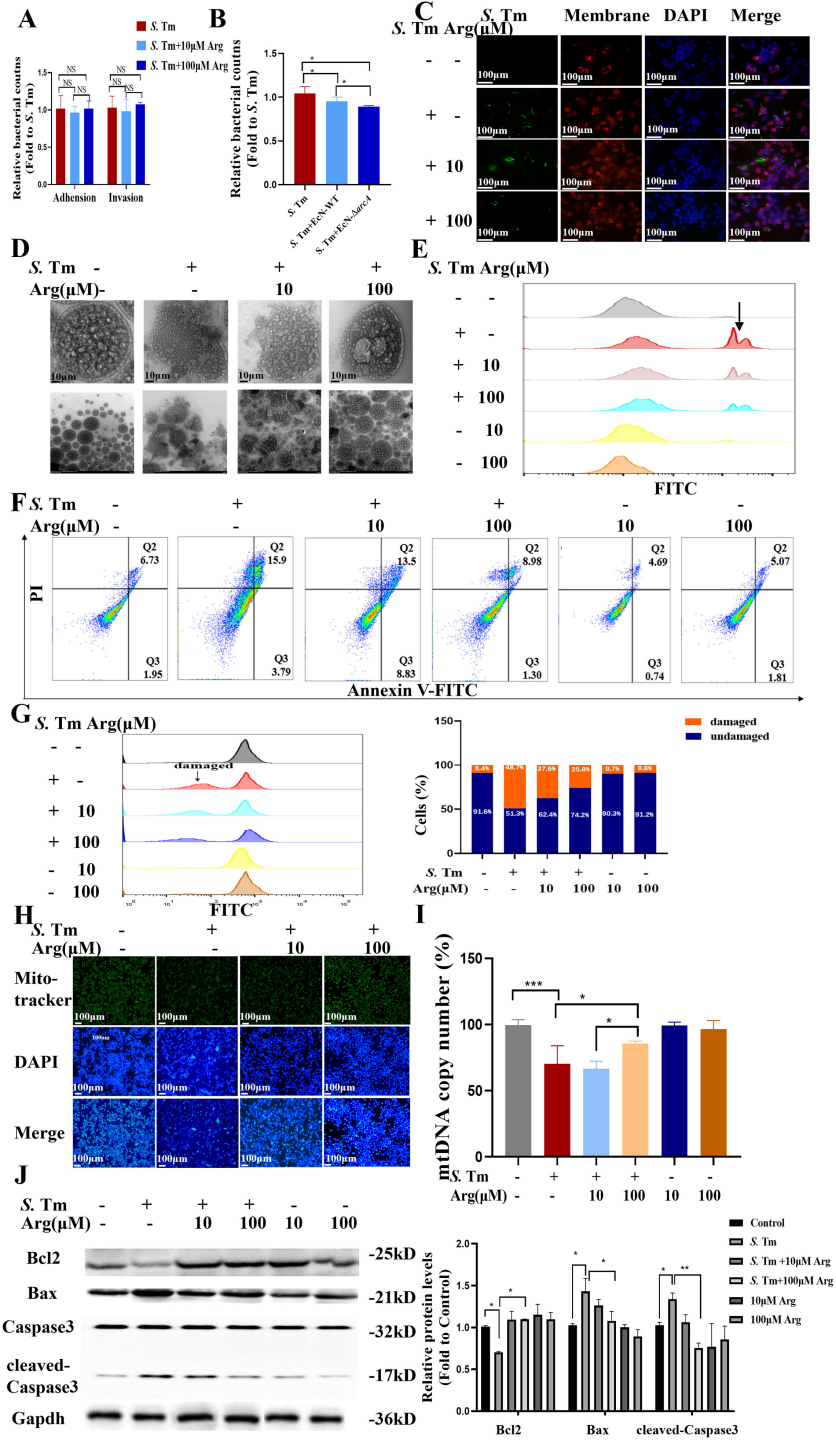
Arginine inhibits macrophage apoptosis **(A)** Adhesion and invasion of bacteria in RAW264.7 cells (*n* = 5 independent experiments). **(B)** Bacteriostatic rates of RAW264.7 cells against *S.* Tm (*n* = 5 independent experiments). **(C)** The IF staining of RAW264.7 cells from each group stained with cytomembrane (Red-Rhodamine), *S.* Tm-EGFP (green), and nuclear (blue) (*n* = 5 independent experiments). **(D)** Observation of cellular morphology by TEM (*n* = 5 independent experiments). **(E)** DCFH-DA fluorescent probe assay for detection of cellular ROS Levels (*n* = 5 independent experiments). **(F)** Representative flow cytometry analysis of Annexin V FITC/PI staining (*n* = 5 independent experiments). **(G)** Representative flow cytometry analysis of Flow cytometry with PI staining for detection and quantification of intracellular DNA content (*n* = 5 independent experiments). **(H)** Cellular mitochondria were stained with Mito-Tracker Green (*n* = 5 independent experiments). **(I)** mtDNA copy number was quantified by qPCR (*n* = 5 independent experiments).**(J)** Western blot of pro and anti-apoptotic proteins, Gapdh was used as housekeeping control (*n* = 5 independent experiments). The data are reported as the mean ± SD. NS, not significant differences; *p < 0.05; **p < 0.01; ***p < 0.001.

To investigate whether arginine directly contributes to macrophage defense against *S*. Tm infection, we treated macrophages with exogenous arginine. Consistent with previous findings, after arginine pretreatment, there was no significant change in the adhesion and invasion of *S.* Tm to RAW264.7 cells (**Figure 5A**). After pretreatment with arginine, the number of extracellular *S.* Tm was significantly reduced following infection of the cells (**Figure 5B**). Using EGFP-labeled *S*. Tm, fluorescence microscopy revealed significantly reduced bacterial loads in arginine-treated macrophage (**Figure 5C**). Transmission electron microscopy demonstrated that arginine treatment preserved mitochondrial ultrastructure, preventing the characteristic swelling, cristae disruption, and vacuolization observed in infected controls (**Figure 5D**).

Quantitative reactive oxygen species (ROS) measurement using 2’,7’-Dichlorodihydrofluorescein diacetate (DCFDA) showed a dose-dependent reduction in *S*. Tm-induced oxidative stress with arginine treatment, with corresponding decreases in fluorescence intensity (**Figure 5E**). Flow cytometric analysis confirmed that arginine supplementation markedly decreased macrophage apoptosis rates during infection (**Figure 5F**). PI staining coupled with RNase treatment revealed that arginine protected against infection-induced DNA degradation (**Figure 5G**).

We further stained the cells using Mito-Tracker Green and evaluated the activity and quantity of mitochondria by the intensity of the fluorescence signal. Mito-Tracker Green staining revealed that arginine treatment prevented *S*. Tm*-*induced reduction in mitochondrial mass (**Figure 5H**). The detection of mitochondrial DNA (mtDNA) copy number is an important means to evaluate mitochondrial function and cellular metabolic status. Quantitative analysis of mtDNA copy number further demonstrated that arginine supplementation dose-dependently attenuated the infection-mediated depletion of mitochondrial genomes (**Figure 5I**). Western blot analysis confirmed that arginine inhibited activation of the mitochondrial apoptosis pathway in *S.* Tm-infected cells (**Figure 5J**). Our results demonstrate that arginine makes a positive contribution to inhibiting the apoptotic process of macrophages under conditions of *S.* Tm challenge, indicating its protective role during bacterial infection.

### Arginine inhibits *S*. Tm-induced apoptosis in murine intestinal macrophages

We next evaluated the protective effects of arginine in *Salmonella*-challenged mice. Intraperitoneal administration of arginine (5 or 50 mg/mL) showed no significant differences in body weight, indicating minimal toxicity at these doses.

**Figure 6:**
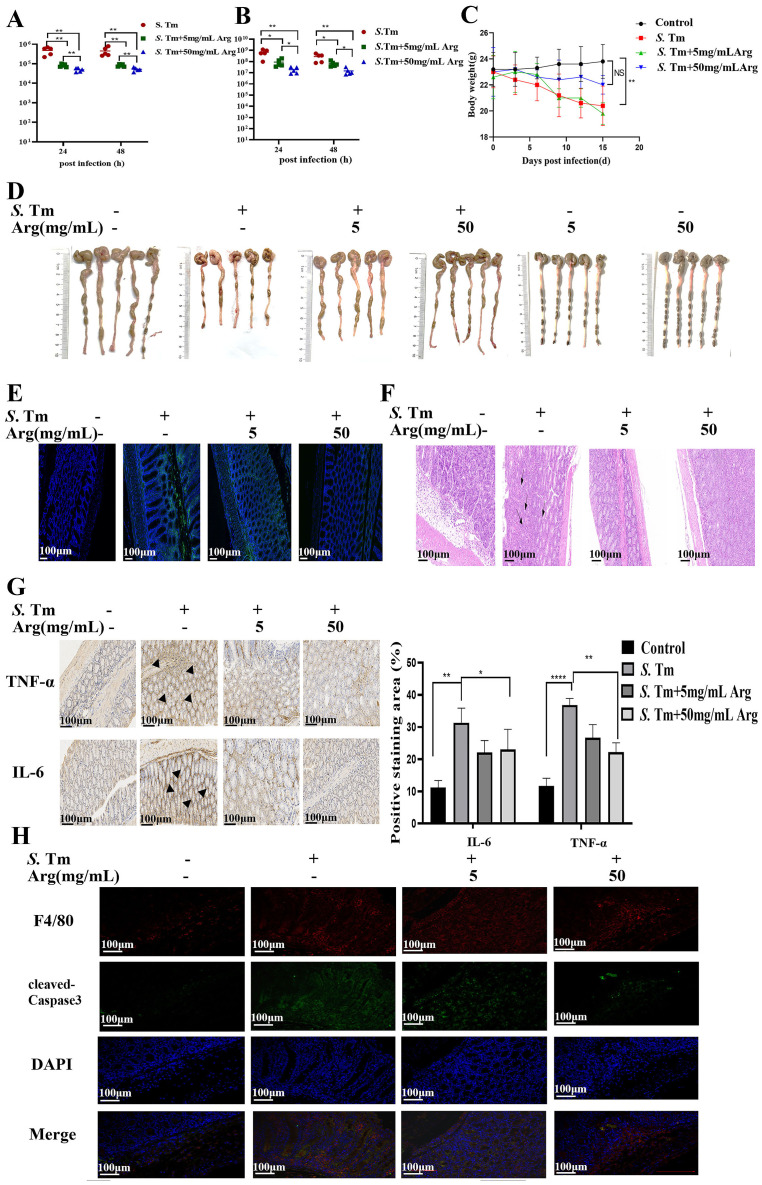
Arginine inhibits S. Tm-induced apoptosis of mouse intestinal macrophages **(A)** Colonic bacterial load among different groups following primary *S.* Tm infection (*n* = 5 mice per group). **(B)** Colonic bacterial load among different groups following consecutive *S.* Tm infection (*n* = 5 mice per group). **(C)** Body weight changes among different groups (*n* = 5 mice per group). **(D)** Representative fecal and colon lengths measured from each group (*n* = 5 mice per group). **(E)** Colonization of *S.* Tm-EGFP in the gut of mice (*n* = 5 mice per group). **(F)** Representative H&E staining of colon sections from different treatment groups (*n* = 5 mice per group). **(G)** IHC was performed to detect IL-6 and TNF-α expression in the indicated colon samples (*n* = 5 mice per group). **(H)** The immunofluorescent images of colon tissues from each group stained with F4/80 (red), cleaved-Caspase3 (green), and nuclear (blue) (*n* = 5 mice per group). The data are reported as the mean ± SD. NS, not significant differences; *p < 0.05; **p < 0.01; ****p < 0.0001.

Quantification of bacterial loads in colon homogenates revealed significantly reduced *S*. Tm colonization in arginine-treated mice under both single (**Figure 6A**) and continuous infection conditions (**Figure 6B**). To delineate arginine’s prophylactic potential, we characterized its impact on survival strategies in mice during sustained *S.* Tm infection. Arginine pretreatment prevented infection-induced weight loss (**Figure 6C**). Morphological measurements showed that *Salmonella* infection resulted in a significant shortening of intestinal length in mice, and arginine intervention could alleviate this pathological change in a dose-dependent manner (**Figure 6D**). Similarly, frozen sections of the intestinal tissue of mice with pEGFP fluorescent plasmid *S*. Tm were observed by in administration, and the results showed that the intestinal fluorescence intensity of mice in the arginine treatment group was lower, indicating that arginine treatment could effectively mitigate *S.* Tm intestinal load (**Figure 6E**). H&E staining analysis demonstrated that arginine treatment significantly preserved mucosal architectural integrity and reduced inflammatory cell infiltration in *S*. Tm-infected intestines (**Figure 6F**). IHC staining revealed that intraperitoneal arginine administration suppressed infection-induced upregulation of proinflammatory cytokines IL-6 and TNF-α (**Figure 6G**). Furthermore, immunofluorescence analysis showed decreased expression of cleaved caspase-3 in colonic macrophages from arginine-treated mice (**Figure 6H**). Collectively, these findings suggest that arginine exert a protective effect against *S*. Tm infection.

### Arginine inhibits macrophage apoptosis by targeting RPS3

**Figure 7:**
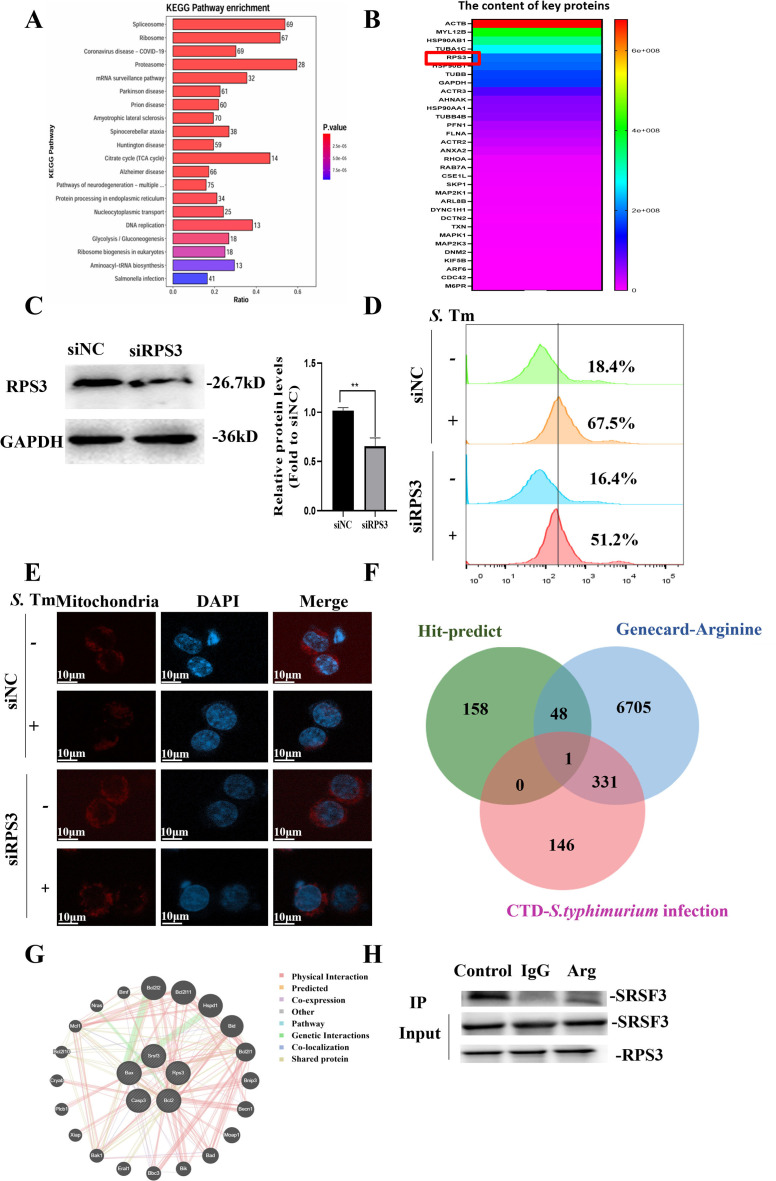
Arginine inhibits *S.* Typhimurium infection by interacting with RPS3 **(-B)** Enrichment analysis of RAW264.7 cells cell lysate and arginine binding protein , pathway **(A)** and heatmap of protein abundance associated with *Salmonella* infection (**B, C**) Knockdown efficiency of RPS3 in RAW264.7 cell line was verified by Western blotting (*n* = 5 independent experiments). **(D)** DCFH-DA fluorescent probe assay for detection of cellular ROS Levels (*n* = 5 independent experiments). **(E)** Representative confocal microscopy image illustrating the mitochondrial abundance within macrophages (*n* = 5 independent experiments). **(F)** Venn diagram of RPS3 binding, arginine metabolism, and protein overlap associated with *Salmonella* infection. **(G)** Interactions between RPS3 interaction with SRSF3 was analyzed using the GeneMANIA database. **(H)** RPS3 and SRSF3 by co-immunoprecipitation assays using anti-SRSF3 antibodies. The data are reported as the mean ± SD. **p < 0.01.

To better understand whether arginine enhances the defense ability of macrophages against *S*. Tm, we lysed the RAW264.7 cells and then incubated them together with biotin-labeled arginine, combined with streptavidin magnetic beads to pull down the biotin-labeled arginine and its bound proteins, and identified them by mass spectrometry (**Figure 7A**). Kyoto Encyclopedia of Genes and Genomes (KEGG) enrichment analysis showed that 41 arginine-binding proteins responded to *Salmonella* infection. Enrichment analysis of these 41 genes showed that, apart from cell membrane proteins, ribosomal protein S3 (RPS3) had a strong interaction with arginine (**Figure 7B**). We further constructed transiently knocked-down cell lines to observe the regulation of RPS3 on *S*. Tm infection (**Figure 7C**).

The results showed that after RPS3 knockdown, the production of ROS in macrophages induced by *S*. Tm decreased (**Figure 7D**), and the reduction in the number of mitochondria caused by *Salmonella* infection was inhibited (**Figure 7E**), suggesting that RPS3 may play an important role in the defense against *S.* Tm infection.

To clarify the function of RPS3 in arginine-enhanced host defense against *S*. Tm infection, we conducted an intersection analysis of the potential binding sites of RPS3, arginine metabolism, and *Salmonella* infection-related proteins. The results showed that there is an interaction between RPS3 and serine/arginine-rich splicing factor 3 (SRSF3) (**Figure 7F**). The results of GENEMANIA analysis indicated that RPS3 and SRSF3 might interact with the key proteins in the mitochondrial apoptosis pathway (**Figure 7G**), suggesting that arginine may inhibit the mitochondrial apoptosis pathway by affecting the interaction between RPS3 and SRSF3.

We then verified the interaction between RPS3 and SRSF3 at the cellular level, and the co-immunoprecipitation (Co-IP) assays in RAW264.7 cell lines demonstrated that RPS3 and SRSF3 exhibit a physiological interaction in cells, and this interaction can be effectively blocked by arginine. (**Figure 7H**). Previous studies have shown that SRSF3 can inhibit apoptosis by binding to the precursor mRNA of BCL-2 interacting cell death suppressor (BIS) (37, 38). Taken together, the above data indicated that during *S.* Tm infection, arginine acts as a critical regulator that stabilizing the cellular state by modulating the interaction between RPS3 and SRSF3.

## Discussion

This study reveals a new mechanism by which *Escherichia coli* Nissle 1917 (EcN) alleviates intestinal inflammation through arginine-mediated suppression of *Salmonella* Typhimurium (*S.* Tm)-induced macrophage apoptosis. Our research results emphasize the crucial role of arginine metabolism in reshaping the “probiotic-pathogen-host” triadic relationship, providing a metabolic perspective interpretation of the therapeutic potential of EcN.

EcN can reduce the adhesion and invasion of *S.* Tm to RAW264.7 cell, but this effect is not related to the intracellular accumulation of arginine. Instead, it may be associated with mechanisms such as competitive exclusion, regulation of intestinal barrier function, modulation of innate immune responses, and production of non-arginine metabolites (32, 39). Arginine accumulation does not affect bacterial adhesion-invasion but may modulate macrophage damage to influence post-invasion pathogenicity, making its investigation essential for understanding effector-phase mechanisms and arginine’s full role in bacterial pathogenesis. The accumulation of arginine promotes macrophage-mediated clearance of extracellular bacteria, which may be associated with mechanisms such as the activation of arginine metabolism to generate antimicrobial substance, regulation of immune signaling pathways to enhance cytokine secretion, remodeling of the extracellular environment to inhibit bacterial proliferation, and promotion of antimicrobial peptide release. *In vivo* experiments demonstrated that EcN/arginine pretreatment can reduce the initial colonization of *S.* Tm, which may be attributed to the synergistic regulation of multiple pathways, such as the dynamic remodeling of immune system, the multicellular network in the intestinal microenvironment and cooperative adaptation of gut microbiota communities.

Although probiotics such as lactobacilli and bifidobacteria mainly act through Toll-like receptor signaling (40, 41) or short-chain fatty acid regulation of immunity (42), EcN inhibits macrophage apoptosis by protecting them from membrane damage, chromatin condensation, and DNA degradation. The uniqueness of EcN lies in its dependence on the arginine accumulation to regulate host-pathogen interaction. This metabolic strategy not only compensates for arginine depletion during *S*. Tm infection but also buffers the inflammatory microenvironment. This mechanism is less emphasized in other probiotics. Our research is consistent with Zhao, who pointed out that the arginine deiminase pathway is a unique feature of EcN compared to *Escherichia coli* BL21 or MG1655, confirming the evolutionary adaptability of amino acid metabolism in EcN’s colonization of the intestine and host defense (43).

The role of arginine in inflammation is dualistic - it can be pro-inflammatory (44, 45) or anti-inflammatory (46, 47). This has always been controversial. Our data support its anti-inflammatory effect, demonstrating that arginine can inhibit ROS, mitochondrial dysfunction, and Caspase-3 activation. This indicates that the action of arginine is environment-dependent, possibly depending on the pH of the inflammatory microenvironment or oxidative stress levels. Arginine acts as a “metabolic checkpoint” through metabolic reprogramming, and compared to directly regulating immune factors, it may reduce the risk of systemic immunosuppression. Combined with related studies, arginine metabolism has cross-interactions with tryptophan and glutamate metabolism (48, 49), suggesting that “imbalance in the amino acid metabolism network” may be a common feature of intestinal inflammation, and EcN plays a core regulatory role in reshaping arginine metabolism. It is notable that the reduction of serum arginine during *S*. Tm infection is similar to the observations in Crohn’s disease, suggesting that arginine depletion may be one of the markers of intestinal flora imbalance. Moreover, exogenous arginine intervention in macrophages can reduce bacterial load in a dose-dependent manner. This indicates that macrophages adopt the “arginine accumulation” strategy to limit the growth of *S*. Tm - during infection, *S*. Tm needs to consume host arginine to promote its own proliferation (e.g., assembling the III-type secretion system) (50, 51), and the continuously released arginine by EcN compensates for this critical resource deficiency.

Further research has found that arginine may inhibit the mitochondrial apoptotic pathway by interfering with the interaction between ribosomal protein S3 (RPS3) and serine/arginine-rich splicing factor 3 (SRSF3). Based on the analysis results from GeneMANIA, RPS3, as a ribosomal protein, plays a non-classical role in apoptosis, which contrasts with the functions of other ribosomal proteins (such as RPS6) in inflammation (52, 53), proposing a new perspective of “auxiliary functions of ribosomal proteins”. Referring to the binding mechanism of SRSF3 with BIS mRNA in related studies, it is speculated that arginine may relieve the isolation of RPS3 from SRSF3 through conformational changes, thereby releasing SRSF3’s regulation of BCL-2 (37). This also implies that in response to bacterial assault, RPS3 might utilize alternative mechanisms to safeguard cells, such as directly engaging in ribosomal stress responses or modulating additional signaling molecules.

In terms of clinical translation, the findings of this study have significant potential application value, but also face some challenges. It is worth noting that arginine can inhibit the function of immune cells and promote tumor development in the tumor microenvironment (54, 55), so the applicability of EcN in patients with co-morbidity of inflammation and tumors needs to be discussed, or it can be considered to be used in combination with arginase inhibitors. Additionally, a key limitation of the current work lies in the lack of *in vivo* validation of arginine concentrations. As highlighted, measuring arginine levels in plasma and intestinal tissues before and after EcN administration would be critical to bridging our *in vitro* findings with *in vivo* relevance. Such data would directly confirm whether the administered EcN dose achieves effective arginine concentrations comparable to those used in our cellular experiments—an essential link for verifying that the observed *in vivo* phenotypes are indeed mediated by arginine elevation. Furthermore, regarding whether arginine exerts its regulatory effect by reducing the binding affinity between RPS3 and SRSF3, direct evidence of molecular interaction is required for support. Direct verification of protein-protein interactions is pivotal to deciphering molecular regulatory pathways. In this field, microscale thermophoresis (MST) and surface plasmon resonance (SPR) are recognized as the gold-standard techniques for quantifying protein-protein interactions. These approaches enable the direct validation of altered binding affinity by precisely measuring the dissociation constant (Kd) of the RPS3-SRSF3 interaction in the presence versus absence of arginine. Integrating these *in vitro* biochemical findings with functional assays in cellular models will facilitate the construction of a complete regulatory map from molecular mechanisms to biological functions, thereby enabling a more comprehensive understanding of the physiological and pathological significance of arginine in relevant signaling pathways or cellular processes.

Nevertheless, this study has for the first time revealed a novel mechanism by which EcN inhibits macrophage apoptosis through arginine production upon *S.* Tm, providing new targets and strategies for the treatment of intestinal inflammation. Future research can focus on the interaction between arginine metabolism and other amino acid metabolism, the specific regulatory mechanism of the interaction between RPS3 and SRSF3, whether arginine regulates the structure of the intestinal microbiota, or affects the balance of regulatory T cells/Th17 cells to reshape the mucosal immune microenvironment, thereby indirectly enhancing the host’s defense ability, and further improving the mechanism map of this triple interaction network to comprehensively reveal the pathogenesis and therapeutic targets of intestinal inflammation.

## Data Availability

The original contributions presented in the study are included in the article/supplementary material. Further inquiries can be directed to the corresponding authors.

## References

[1] Gilliland A Chan JJ De Wolfe TJ Yang H Vallance BA. Pathobionts in inflammatory bowel disease: origins, underlying mechanisms, and implications for clinical care. Gastroenterology. (2024) 166:44–58. doi: 10.1053/j.gastro.2023.09.019, PMID: 37734419

[2] Wu T Cheng H Zhuang J Liu X Ouyang Z Qian R. Risk factors for inflammatory bowel disease: an umbrella review. Front Cell Infect Microbiol. (2025) 14:1410506. doi: 10.3389/fcimb.2024.1410506, PMID: 39926114 PMC11802543

[3] Khor B Gardet A Xavier RJ. Genetics and pathogenesis of inflammatory bowel disease. Nature. (2011) 474:307–17. doi: 10.1038/nature10209, PMID: 21677747 PMC3204665

[4] Kumbhari A Cheng TNH Ananthakrishnan AN Kochar B Burke KE Shannon K. Discovery of disease-adapted bacterial lineages in inflammatory bowel diseases. Cell Host Microbe. (2024) 32:1147–1162.e12. doi: 10.1016/j.chom.2024.05.022, PMID: 38917808 PMC11239293

[5] Zhang YZ Li YY. Inflammatory bowel disease: pathogenesis. World J Gastroenterol. (2014) 20:91–9. doi: 10.3748/wjg.v20.i1.9 PMC3886036 24415861

[6] Qiu P Ishimoto T Fu L Zhang J Zhang Z Liu Y. The gut microbiota in inflammatory bowel disease. Front Cell Infect Microbiol. (2022) 12:733992. doi: 10.3389/fcimb.2022.733992, PMID: 35273921 PMC8902753

[7] Caruso R Lo BC Núñez G. Host-microbiota interactions in inflammatory bowel disease. Nat Rev Immunol. (2020) 20:411–26. doi: 10.1038/s41577-019-0268-7, PMID: 32005980

[8] Cherrak Y Younes AA Perez-Molphe-Montoya E Maurer L Yilmaz K Enz U. Neutrophil recruitment during intestinal inflammation primes Salmonella elimination by commensal E. coli in a context-dependent manner. Cell Host Microbe. (2025) 33:358–372.e4. doi: 10.1016/j.chom.2025.02.004, PMID: 40023150

[9] Smith AB Jenior ML Keenan O Hart JL Specker J Abbas A. Enterococci enhance Clostridioides difficile pathogenesis. Nature. (2022) 611:780–6. doi: 10.1038/s41586-022-05438-x, PMID: 36385534 PMC9691601

[10] Bäumler AJ Sperandio V. Interactions between the microbiota and pathogenic bacteria in the gut. Nature. (2016) 535:85–93. doi: 10.1038/nature18849, PMID: 27383983 PMC5114849

[11] Herzog MK Cazzaniga M Peters A Shayya N Beldi L Hapfelmeier S. Mouse models for bacterial enteropathogen infections: insights into the role of colonization resistance. Gut Microbes. (2023) 15:2172667. doi: 10.1080/19490976.2023.2172667, PMID: 36794831 PMC9980611

[12] Jess T Simonsen J Nielsen NM Jørgensen KT Bager P Ethelberg S. Enteric Salmonella or Campylobacter infections and the risk of inflammatory bowel disease. Gut. (2011) 60:318–24. doi: 10.1136/gut.2010.223396, PMID: 21193449

[13] Alpuche-Aranda CM Racoosin EL Swanson JA Miller. Salmonella stimulate macrophage macropinocytosis SI. and persist within spacious phagosomes. J Exp Med. (1994) 179:601–8. doi: 10.1084/jem.179.2.601, PMID: 8294870 PMC2191354

[14] Schütz E. The treatment of intestinal diseases with Mutaflor. A multicenter retrospective study. Fortschr Med. (1989) 107:599–602., PMID: 2693288

[15] Schultz M. Clinical use of E. coli Nissle 1917 in inflammatory bowel disease. Inflammation Bowel Dis. (2008) 14:1012–8. doi: 10.1002/ibd.20377, PMID: 18240278

[16] Jacobi CA Malfertheiner P. Escherichia coli Nissle 1917 (Mutaflor): new insights into an old probiotic bacterium. Dig Dis. (2011) 29:600–7. doi: 10.1159/000333307, PMID: 22179217

[17] Zhao Z Xu S Zhang W Wu D Yang G. Probiotic Escherichia coli NISSLE 1917 for inflammatory bowel disease applications. Food Funct. (2022) 13:5914–24. doi: 10.1039/d2fo00226d, PMID: 35583304

[18] Scaldaferri F Gerardi V Mangiola F Lopetuso LR Pizzoferrato M Petito V. Role and mechanisms of action of Escherichia coli Nissle 1917 in the maintenance of remission in ulcerative colitis patients: An update. World J Gastroenterol. (2016) 22:5505–11. doi: 10.3748/wjg.v22.i24.5505, PMID: 27350728 PMC4917610

[19] Agus A Planchais J Sokol H. Gut microbiota regulation of tryptophan metabolism in health and disease. Cell Host Microbe. (2018) 23:716–24. doi: 10.1016/j.chom.2018.05.003, PMID: 29902437

[20] Lavelle A Sokol H. Gut microbiota-derived metabolites as key actors in inflammatory bowel disease. Nat Rev Gastroenterol Hepatol. (2020) 17:223–37. doi: 10.1038/s41575-019-0258-z, PMID: 32076145

[21] Li JY Guo YC Zhou HF Yue TT Wang FX Sun F. Arginine metabolism regulates the pathogenesis of inflammatory bowel disease. Nutr Rev. (2023) 81:578–86. doi: 10.1093/nutrit/nuac070, PMID: 36040377 PMC10086623

[22] Langston C. Managing fluid and electrolyte disorders in kidney disease. Vet Clin North Am Small Anim Pract. (2017) 47:471–90. doi: 10.1016/j.cvsm.2016.09.011, PMID: 27908485

[23] De Backer D. Lactic acidosis. Intensive Care Med. (2003) 29:699–702. doi: 10.1007/s00134-003-1746-7, PMID: 12682722

[24] Pugin J Dunn-Siegrist I Dufour J Tissières P Charles PE Comte R. Cyclic stretch of human lung cells induces an acidification and promotes bacterial growth. Am J Respir Cell Mol Biol. (2008) 38:362–70. doi: 10.1165/rcmb.2007-0114OC, PMID: 17921360

[25] Xu X Ocansey DKW Pei B Zhang Y Wang N Wang Z. Resveratrol alleviates DSS-induced IBD in mice by regulating the intestinal microbiota-macrophage-arginine metabolism axis. Eur J Med Res. (2023) 28:319. doi: 10.1186/s40001-023-01257-6, PMID: 37660064 PMC10474707

[26] Lee CA Falkow S. The ability of Salmonella to enter mammalian cells is affected by bacterial growth state. Proc Natl Acad Sci U S A. (1990) 87:4304–8. doi: 10.1073/pnas.87.11.4304, PMID: 2349239 PMC54097

[27] Wu J Pugh R Laughlin RC Andrews-Polymenis H McClelland M Bäumler AJ. High-throughput assay to phenotype Salmonella enterica typhimurium association, invasion, and replication in macrophages. J Vis Exp. (2014) 90:e51759. doi: 10.3791/51759, PMID: 25146526 PMC4500590

[28] Jiang Y Chen B Duan C Sun B Yang J Yang S. Multigene editing in the Escherichia coli genome via the CRISPR-Cas9 system. Appl Environ Microbiol. (2015) 81:2506–14. doi: 10.1128/AEM.04023-14, PMID: 25636838 PMC4357945

[29] Keng VW Watson AL Rahrmann EP Li H Tschida BR Moriarity BS. Conditional inactivation of pten with EGFR overexpression in schwann cells models sporadic MPNST. Sarcoma. (2012) 2012:620834. doi: 10.1155/2012/620834, PMID: 23319880 PMC3539440

[30] Warde-Farley D Donaldson SL Comes O Zuberi K Badrawi R Chao P. The GeneMANIA prediction server: biological network integration for gene prioritization and predicting gene function. Nucleic Acids Res. (2010) 38:W214–20. doi: 10.1093/nar/gkq537, PMID: 20576703 PMC2896186

[31] Patil A Nakai K Nakamura H. HitPredict: a database of quality assessed protein-protein interactions in nine species. Nucleic Acids Res. (2011) 39:D744–9. doi: 10.1093/nar/gkq897, PMID: 20947562 PMC3013773

[32] Deriu E Liu JZ Pezeshki M Edwards RA Ochoa RJ Contreras H. Probiotic bacteria reduce salmonella typhimurium intestinal colonization by competing for iron. Cell Host Microbe. (2013) 14:26–37. doi: 10.1016/j.chom.2013.06.007, PMID: 23870311 PMC3752295

[33] Cheng WY Tong H Miller EW Chang CJ Remington J Zucker RM. An integrated imaging approach to the study of oxidative stress generation by mitochondrial dysfunction in living cells. Environ Health Perspect. (2010) 118:902–8. doi: 10.1289/ehp.0901811, PMID: 20413366 PMC2920907

[34] Barthel M Hapfelmeier S Quintanilla-Martínez L Kremer M Rohde M Hogardt M. Pretreatment of mice with streptomycin provides a Salmonella enterica serovar Typhimurium colitis model that allows analysis of both pathogen and host. Infect Immun. (2003) 71:2839–58. doi: 10.1128/IAI.71.5.2839-2858.2003, PMID: 12704158 PMC153285

[35] Xiong D Song L Chen Y Jiao X Pan Z. Salmonella Enteritidis activates inflammatory storm via SPI-1 and SPI-2 to promote intracellular proliferation and bacterial virulence. Front Cell Infect Microbiol. (2023) 13:1158888. doi: 10.3389/fcimb.2023.1158888, PMID: 37325511 PMC10266283

[36] Cui X Wang Y Liu J Liu Z Zhao M Yu W. Dietary limonin alleviates Salmonella Typhimurium-induced colitis via dual targeting virulence SopB and SopE2 and inhibiting RAC1/CDC42/Arp2/3 pathway and regulating gut microbiota. Food Funct. (2025) 16:1041–59. doi: 10.1039/d4fo02810d, PMID: 39820212

[37] Baek JY Yun HH Jung SY Lee J Yoo K Lee JH. SRSF3 is a critical requirement for inclusion of exon 3 of BIS pre-mRNA. Cells. (2020) 9:2325. doi: 10.3390/cells9102325, PMID: 33086735 PMC7589869

[38] Lee JH Takahashi T Yasuhara N Inazawa J Kamada S Tsujimoto Y. Bis, a Bcl-2-binding protein that synergizes with Bcl-2 in preventing cell death. Oncogene. (1999) 18:6183–90. doi: 10.1038/sj.onc.1203043, PMID: 10597216

[39] Patzer SI Baquero MR Bravo D Moreno F Hantke K The colicin G. H and X determinants encode microcins M and H47, which might utilize the catecholate siderophore receptors FepA, Cir, Fiu and IroN. Microbiology. (2003) 149:2557–70. doi: 10.1099/mic.0.26396-0, PMID: 12949180

[40] Koh JA Le MH Lee HJ Min JW Kim MJ Kang HC. Extracellular vesicles derived from Lactobacillus gasseri GFC-1220 alleviate inflammation via the TLR4/NF-κB signaling pathway in LPS-stimulated RAW264.7 macrophages. Sci Rep. (2025) 15:21381. doi: 10.1038/s41598-025-99160-z, PMID: 40595215 PMC12215889

[41] Chen S Shi M Chen X Le Q He J. Lactiplantibacillus plantarum YDJ-03 and limosilactobacillus fermentum YDJ-6 alleviate metabolic syndrome in mice. Int J Vitam Nutr Res. (2025) 95:31275. doi: 10.31083/IJVNR31275, PMID: 40298159

[42] Tsui Y Wu X Zhang X Peng Y Mok CKP Chan FKL. Short-chain fatty acids in viral infection: the underlying mechanisms, opportunities, and challenges. Trends Microbiol. (2025) 33:302–20. doi: 10.1016/j.tim.2024.10.001, PMID: 39505671

[43] Zhao L Yin G Zhang Y Duan C Wang Y Kang Z. A comparative study on the genomes, transcriptomes, and metabolic properties of Escherichia coli strains Nissle 1917, BL21(DE3), and MG1655. Eng Microbiol. (2022) 2:100012. doi: 10.1016/j.engmic.2022.100012, PMID: 39628614 PMC11610980

[44] Mossmann D Müller C Park S Ryback B Colombi M Ritter N. Arginine reprograms metabolism in liver cancer via RBM39. Cell. (2023) 186:5068–5083.e23. doi: 10.1016/j.cell.2023.09.011, PMID: 37804830 PMC10642370

[45] Asosingh K Lauruschkat CD Alemagno M Frimel M Wanner N Weiss K. Arginine metabolic control of airway inflammation. JCI Insight. (2020) 5:e127801. doi: 10.1172/jci.insight.127801, PMID: 31996482 PMC7098726

[46] Chen MY Sun CY Zhao R Guan XL Li ML Zhang F. BAG2 releases SAMD4B upon sensing of arginine deficiency to promote tumor cell survival. Mol Cell. (2025) 85(13):2581–96.e6. doi: 10.1016/j.molcel.2025.05.035, PMID: 40555234

[47] Cao S Li Y Song R Meng X Fuchs M Liang C. L-arginine metabolism inhibits arthritis and inflammatory bone loss. Ann Rheum Dis. (2024) 83:72–87. doi: 10.1136/ard-2022-223626, PMID: 37775153 PMC10803985

[48] Taubenheim J Kadibalban AS Zimmermann J Taubenheim C Tran F Rosenstiel P. Metabolic modeling reveals a multi-level deregulation of host-microbiome metabolic networks in IBD. Nat Commun. (2025) 16:5120. doi: 10.1038/s41467-025-60233-2, PMID: 40456745 PMC12130198

[49] Leibovitzh H Lee SH Xue M Raygoza Garay JA Hernandez-Rocha C Madsen KL. Altered gut microbiome composition and function are associated with gut barrier dysfunction in healthy relatives of patients with crohn’s disease. Gastroenterology. (2022) 163:1364–1376.e10. doi: 10.1053/j.gastro.2022.07.004, PMID: 35850197

[50] Margolis A Liu L Porwollik S Till JKA Chu W McClelland M. Arginine metabolism powers salmonella resistance to oxidative stress. Infect Immun. (2023) 91:e0012023. doi: 10.1128/iai.00120-23, PMID: 37191509 PMC10269097

[51] Brigo N Neumaier E Pfeifhofer-Obermair C Grubwieser P Engl S Berger S. Timing of Interleukin-4 Stimulation of Macrophages Determines Their Anti-Microbial Activity during Infection with Salmonella enterica Serovar Typhimurium. Cells. (2023) 12:1164. doi: 10.3390/cells12081164, PMID: 37190073 PMC10137269

[52] Özduman G Javed A Akçaöz Alasar A Akgül B Korkmaz KS. HN1 Functions in Protein Synthesis Regulation via mTOR-RPS6 Axis and Maintains Nucleolar Integrity. Cell Prolif. (2025) 58:e13805. doi: 10.1111/cpr.13805, PMID: 39805577 PMC12179552

[53] Liu C Liang N Wu C Qiu Z Huang Q Wei X. Gasdermin D-mediated pyroptosis exerts two opposite effects of resisting enzymatic digestion and expanding inflammatory response in acute pancreatitis. Adv Sci (Weinh). (2025) 29:e02412. doi: 10.1002/advs.202502412, PMID: 40439590 PMC12376566

[54] Zou Z Cheng Q Zhou J Guo C Hadjinicolaou AV Salio M. ATF4-SLC7A11-GSH axis mediates the acquisition of immunosuppressive properties by activated CD4+ T cells in low arginine condition. Cell Rep. (2024) 43:113995. doi: 10.1016/j.celrep.2024.113995, PMID: 38527061

[55] Zhu Y Zhou Z Du X Lin X Liang ZM Chen S. Cancer cell-derived arginine fuels polyamine biosynthesis in tumor-associated macrophages to promote immune evasion. Cancer Cell. (2025) 43:1045–1060.e7. doi: 10.1016/j.ccell.2025.03.015, PMID: 40185095

